# Two surgical strategies for treating multilevel cervical spondylotic myelopathy combined with kyphotic deformity

**DOI:** 10.1097/MD.0000000000019215

**Published:** 2020-02-14

**Authors:** Kuang-Ting Yeh, Ing-Ho Chen, Ru-Ping Lee, Tzai-Chiu Yu, Cheng-Huan Peng, Kuan-Lin Liu, Jen-Hung Wang, Wen-Tien Wu

**Affiliations:** a Department of Orthopedics, Hualien Tzu Chi Hospital, Buddhist Tzu Chi Medical Foundation; b School of Medicine, Tzu Chi University; c Institute Medical Sciences, Tzu Chi University; d Department of Medical Research, Hualien Tzu Chi Hospital, Buddhist Tzu Chi Medical Foundation, Hualien, Taiwan.

**Keywords:** anterior fusion combined with posterior instrumentation, laminoplasty combined with anterior fusion, multilevel cervical spondylotic myelopathy with kyphotic deformity

## Abstract

This study compared the surgical outcomes of two surgical methods for treating multilevel cervical spondylotic myelopathy (MCSM) combined with cervical kyphotic deformity (CKD): (1) the ELTA method consisted of expansive open-door laminoplasty (EOLP) followed by three-segment anterior cervical discectomy fusion (ACDF), and (2) the LAPI method consisted of long-segment ACDF followed by long-level posterior instrumented fusion (PIF). Surgical treatment of CKD combined with MCSM remains challenging. Surgical considerations should include adequate spinal cord decompression and restoration of satisfactory cervical sagittal alignment (CSA). In certain situations, a solid PIF structure is vital to prevent failure.

We included 105 patients who underwent the aforementioned surgical methods for MCSM combined with CKD from January 2013 to December 2017. The minimum follow-up period was 1 year. Comparative analysis was performed to compare the two surgical strategies’ preoperative and postoperative functional outcomes, including a visual analog scale for neck pain, neck disability index, the Japanese Orthopedic Association cervical myelopathy score, and the Nurick score, as well as the CSA radiographic outcomes, including C2-7 Cobb angle, C2-7 sagittal vertical axis, and C7 slope. The risk factors related to reduced improvement in functional status were analyzed.

A total of 63 patients underwent ELTA and 42 patients underwent LAPI. Improvements in functional outcomes were considerable in both groups. The mean C2-7 Cobb angle was restored from 7.4° ± 2.1° kyphosis to 8.8° ± 4.7° lordosis in the ELTA group and from 15.3° ± 4.2° kyphosis to 15.8° ± 8.1° lordosis in the LAPI group. The maximal correction angle was 22.6° in the ELTA group and 42.6° in the LAPI group.

Although changes in CSA seemed to be significantly correlated with improvements of functional status, the ELTA and LAPI methods were both effective for treating MCSM combined with CKD, when appropriately selected. The ELTA method was indicated for MCSM patients who had a low degree of CKD, whereas the LAPI method was indicated for MCSM patients who had poor function scores and a high degree of CKD.

## 1 Introduction

Cervical kyphotic deformity (CKD) adversely affects horizontal gaze, swallowing, and respiration.^[1]^ As a reversal of normal cervical spine curvature, CKD can lead to mechanical neck pain, neurological dysfunction, and functional disabilities^[2]^; furthermore, it can shift the spinal cord to the anterior portion of the spinal canal, which leads to increased mechanical stress on the spinal cord.^[3]^ Multilevel cervical spondylotic myelopathy (MCSM) is often caused by the compression of degenerated facet joints, hypertrophic or ossificated ligamentum flavum, or degenerated bulging disks.^[4]^ When MCSM is combined with CKD, the spinal cord can be compressed circumferentially. The small anterior feeding vessels of the spinal cord are flattened during cervical flexion, which can lead to chronic cord ischemia, myelomalacia, and spinal cord atrophy.^[5]^ Patients with CKD and MCSM and who are progressively symptomatic are frequently admitted for surgical intervention. The main goals of surgery are to decompress the spinal cord and correct CKD to neutral or lordotic cervical curvature. Anterior, posterior, and combined approaches are the three main surgery methods.^[6]^ Long-segment anterior cervical discectomy and fusion (ACDF) is a commonly used procedure because it can achieve a considerable degree of correction^[7]^; however, two principal limitations have been reported for long-segment ACDF in treating MCSM combined with CKD. One is that the spinal cord is more easily injured through the anterior approach due to anterior shifting with the posterior elements compressed.^[2]^ The other is that without posterior instrumented fusion (PIF), failure to fuse the corrected segments can occur.^[8]^ Following our previous study, we applied expansive open-door laminoplasty (EOLP) followed by multisegment ACDF to treat MCSM with local kyphosis or major anterior pathology. This was applied to achieve preservation of motion segments and increased safety for the anterior procedure through posterior shifting of the spinal cord by laminoplasty.^[9]^ EOLP with short-segment PIF using lateral mass screws is beneficial for patients with MCSM with short-segment instability but without fixed CKD.^[10]^ For patients with CKD with higher degrees of kyphotic deformity, circumferential decompression and fusion is a common surgical treatment option that often includes long-segment ACDF followed by PIF (with lateral mass screws or pedicle screws) and posterior decompression (PD) (with laminectomy or laminoplasty).^[11,12]^ Although this combined approach is thought to provide a greater mechanical advantage for deformity correction, our previous study indicated that it may carry higher mortality and morbidity rates. The present study presented the surgical results of the two different operation strategies for patients who were diagnosed as having MCSM combined with CKD.

## 2 Materials and methods

This study was approved by the Research Ethics Committee of Hualien Tzu Chi Hospital, Buddhist Tzu Chi Medical Foundation, under the professional confirmation of a radiologist (IRB103-189-B). All patients provided informed consent. We collected and retrospectively reviewed data from patients who were diagnosed as having MCSM (involving more than three motion segments) combined with CKD (a Cobb angle between C2 and C7 indicated kyphotic deformity of more than 5°) and had received cervical spinal correction and fusion surgery from January 2013 to December 2017. Two surgical strategies were employed:

(1) EOLP followed by three-segment ACDF (simplified as the ELTA method) and
(2) long-segment ACDF followed by PIF and PD (simplified as the LAPI method).

Patients who had concomitant major thoracolumbar spinal pathology or who had no follow-up data were excluded. The ELTA method involved C3-6 EOLP secured with miniplates (Synthes, MI) and a C7 partial laminectomy^[13]^ followed by C3-6 or C4-7 ACDFs with titanium cages. The LAPI method involved applying ACDFs (>3 segments) with polyetheretherketone (PEEK) cages, C2-7 or C3-7 PIF with lateral mass or a pedicle screw-rod system (Synthes Spine, Westchester, PA), and PD through laminoplasty secured by miniplates (Synthes, MI). The anterior and posterior surgeries were both performed on the same day. All patients wore a hard cervical collar for 12 weeks after surgery.

The patients were divided into two groups for comparative analysis based on the surgical strategy they had received. The demographic data analyzed were age, sex, body mass index (BMI), and trauma history before symptom aggravation or before symptoms had been recorded. Clinical outcome evaluations to determine a patient's neurologic deficit status were the Nurick score^[14]^ and Japanese Orthopedic Association (JOA) score^[15]^; to evaluate functional status, the neck disability index (NDI)^[16]^ and visual analog scale (VAS) of neck pain were used. We collected the functional scores before surgery and 12 months postsurgery. Radiographic outcomes evaluated were the C7 slope and C2-7 Cobb angle before surgery and 12 months postsurgery, from neutral lateral plain films. The C2-7 Cobb angle was calculated as the angle between the C2 and C7 lower endplates and the C7 slope was calculated as the angle between the horizontal plane and the C7 superior endplate.^[17]^ We conducted a computed tomography scan 6 months postsurgery to assess each patient's bony fusion status. Magnetic resonance imaging (MRI) was performed 12 months postsurgery to assess each patient's decompression status.

SPSS version 17.0 was used for statistical analysis. The categorical variables were analyzed through a chi-squared test, whereas the continuous variables were analyzed through independent *t* tests. Independent *t* tests were conducted to compare the functional parameters of the patients who underwent the two surgical methods. The risk factors of poor functional recovery were analyzed using a generalized linear model (GLM). To assess statistical significance, an unpaired Student *t* test was performed. The level of statistical significance was set as *P* < .05.

## 3 Results

Data collected from 105 patients were analyzed; 63 patients underwent ELTA and 42 underwent LAPI. Of the patients, 54 were men and 51 were women (mean age: 64.5 ± 11.9 years). In terms of BMI, 39 (37.1%) of the participants had a BMI in the normal range and 66 (62.9%) were overweight or obese. Of the participants, eight (7.6%) had diabetes mellitus (DM) and seven (6.7%) had a smoking habit (Table 1). NDI and Nurick scores significantly improved in both groups. The mean JOA score demonstrated a greater improvement in the ELTA group (from 8.5 ± 2.6 to 13.7 ± 2.9) than in the LAPI group (from 12.1 ± 2.1 to 15.9 ± 1.0), whereas the mean VAS score for neck pain demonstrated a greater improvement in the LAPI group (from 5.9 ± 0.8 to 2.8 ± 1.5) than in the ELTA group (from 7.1 ± 2.1 to 2.5 ± 1.3) (Table 2). The mean C2-7 Cobb angle increased from 7.4° ± 2.1° kyphosis to 8.8° ± 4.7° lordosis in the ELTA group and from 15.3° ± 4.2° kyphosis to 15.8° ± 8.1° lordosis in the LAPI group (Table 2). The maximal correction angle was 22.6° in the ELTA group and 42.6° in the LAPI group. The loss of correction at postoperative year 1 was <5° in both groups.

**Table 1:**
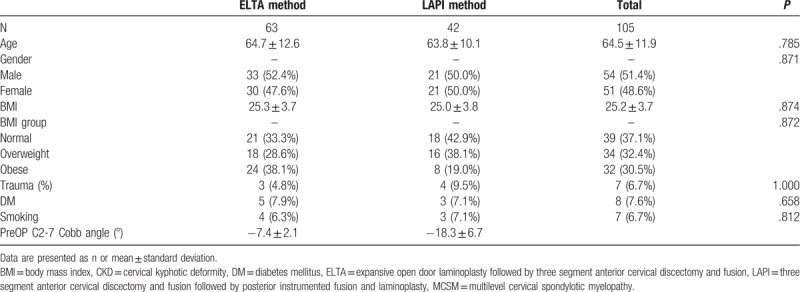
Demographics and function score of the patients diagnosed as MCSM with CKD.

**Table 2:**
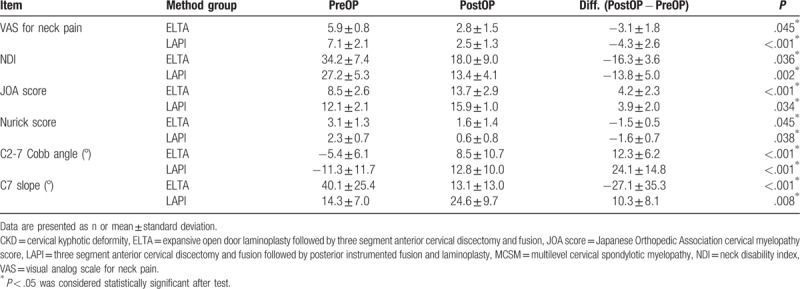
Functional improvement of the patients who were diagnosed as MCSM with CKD and received LAPI or ELTA methods (n = 105).

Correlations of risk factors with improvements in functional status were analyzed using a GLM. The improvement in the VAS score for neck pain was more significant in women, obese patients, and ELTA-treated patients. The improvement of NDI was more significant in women and in the LAPI group. Changes in the C2-7 Cobb angle and the C7 slope were not significantly correlated with differences in functional scores (Table 3).

**Table 3:**
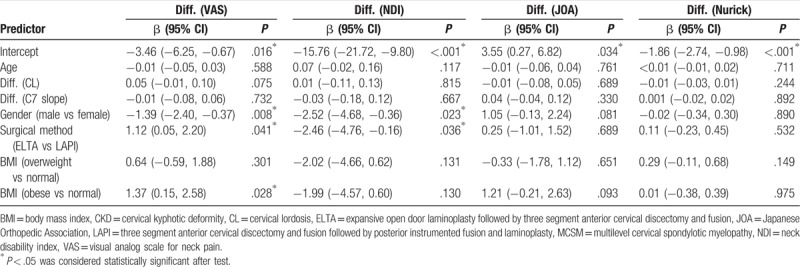
Factors associated with the improvement of function scores of the patients who were diagnosed as MCSM with CKD (n = 87).

Seven of the 105 patients (6.7%) had poor posterior side wound healing and required surgical debridement and repair: four were in the ELTA group (6.3%) and the three were in the LAPI group (7.1%). All 105 patients had mild to moderate dysphagia 2 weeks postsurgery, which persisted in 35 (33.3%) patients 2 to 6 weeks postsurgery: 25 of such patients were in the ELTA group (39.7%) and 10 were in the LAPI group (23.8%). No complications regarding hardware malposition, pseudoarthrosis, neurologic deterioration, aggravated neck pain, C5 nerve palsy, fusion segment collapse, or admission for revision surgery were observed in all the patients included in this study.

### 3.1 ELTA method case report

**Figure 1:**
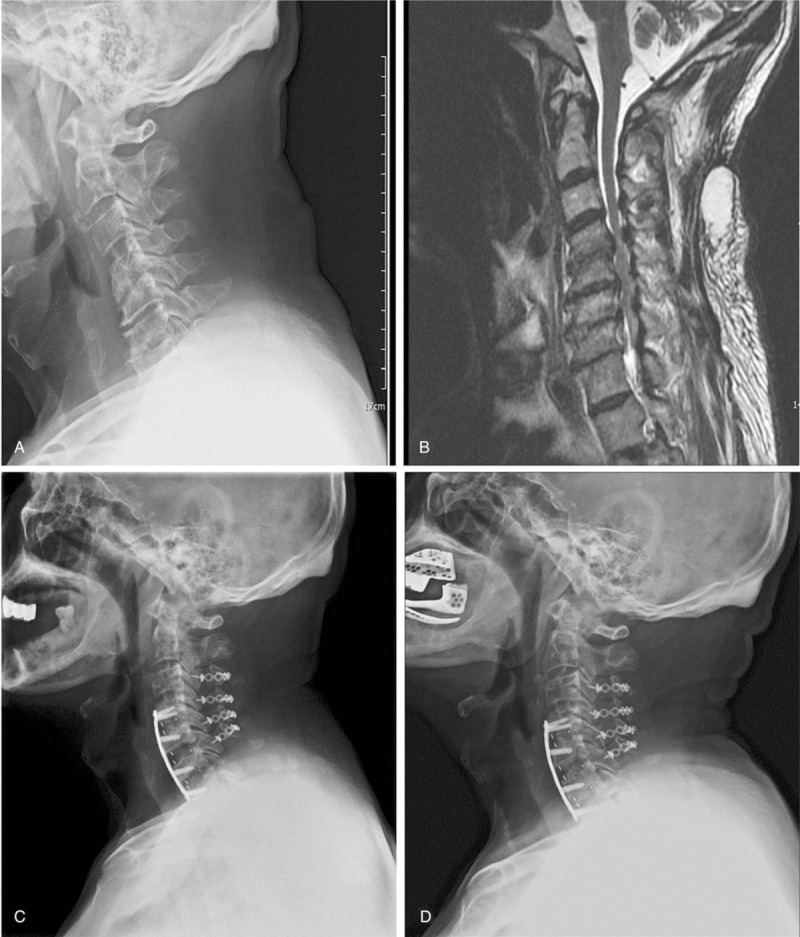
A 65-year-old man underwent C3-6 EOLP and C7 partial laminectomy followed by C4-7 ACDF with PEEK cages. (A) The preoperative plain film demonstrated CKD. (B) Midsagittal T2-weighted MRI revealed C3-7 multilevel stenosis with spinal cord compression. (C) The postoperative 3-month plain film revealed restoration of the cervical lordotic curve and enlargement of the spinal canal. (D) The postoperative 12-month plain film revealed an effective union of the anterior fusion structure. ACDF = anterior cervical discectomy fusion, CKD = cervical kyphotic deformity, EOLP = expansive open-door laminoplasty, MRI = magnetic resonance imaging, PEEK = polyetheretherketone.

A 65-year-old man presented with an unsteady gait, weakness of all four limbs, and aggravated neck pain; these symptoms were experienced for 2 months. Neurologic deficit and reduced life quality were noted. Preoperative plain films revealed the existence of a C2-7 Cobb angle indicating 6° kyphosis and C7 slope representing 25° lordosis (Fig. 1A). MRI revealed C3-7 stenosis and CKD mainly at the C4-7 level (Fig. 1B). The patient underwent C3-6 EOLP and C7 partial laminectomy followed by C4-7 ACDF with PEEK cages (Fig. 1C). His JOA score improved from 10 to 16, his Nurick score decreased from 3 to 1, his NDI decreased from 40 to 20, his neck pain score improved from 6 to 2, and his postoperative C2-7 Cobb angle and C7 slope were 17° lordosis and 29° lordosis, respectively. Lateral plain film 12 months postsurgery revealed a solid union over the anterior fusion segments (Fig. 1D).

### 3.2 LAPI method case report

**Figure 2:**
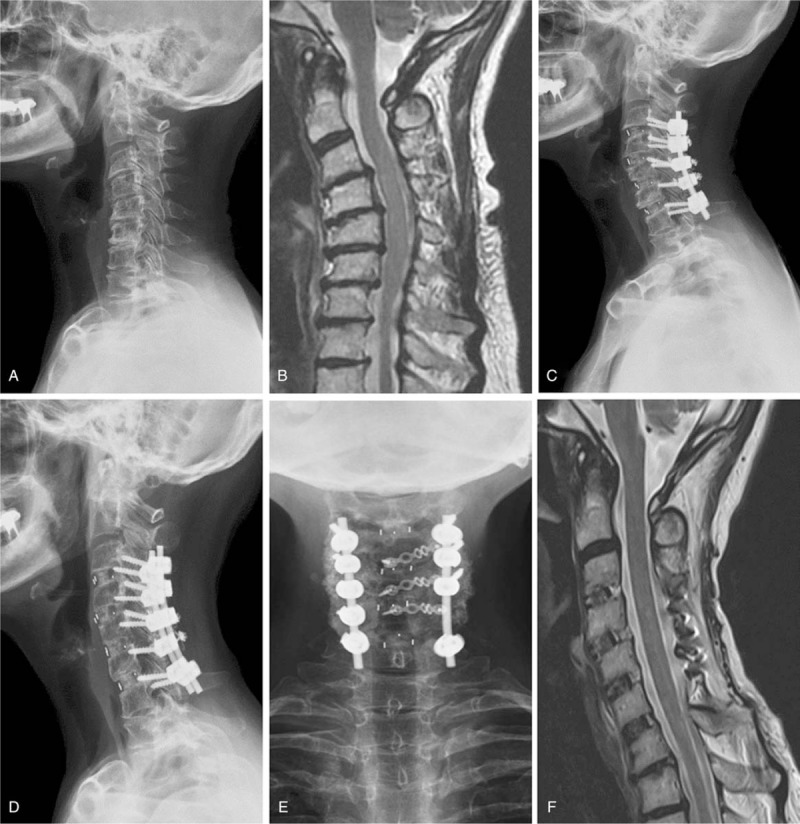
A 66-year-old woman received C3-7 ACDF with PEEK cages, PIF with C3-6 lateral mass screws and C7 pedicle screws, and PD with C3-6 laminoplasty. (A) The preoperative plain film demonstrated 13° cervical kyphosis. (B) Midsagittal T2-weighted MRI revealed spinal cord compression over C3-7 kyphotic stenosis. (C) The postoperative 3-month plain film revealed restoration of the cervical lordotic curve (18° lordosis) with augmented PIF and a chipped bone graft placed over the laminae of the hinge side and bilateral mass. (D, E) The plain films 12 months postsurgery reveal union over the anterior fusion segments and posterior bilateral mass. (F) Midsagittal T2-weighted MRI 12 months postsurgery reveals patent spinal canal over C3-7. ACDF = anterior cervical discectomy fusion, MRI = magnetic resonance imaging, PEEK = polyetheretherketone, PIF = posterior instrumented fusion.

A 66-year-old woman had comorbidities of hypertension, type II DM, and osteoporosis under regular medication control. Aggravated neck pain with bilateral hand clumsiness were noted after the patient fell when walking 1 month prior. Rehabilitation therapy and oral analgesics were unsuccessful. At our outpatient department, positive long tract, and Spurling signs were identified in the patient. Preoperative plain films revealed CKD (Fig. 2A), and MRI revealed C3-7 stenosis with spinal cord compression (Fig. 2B). The patient received C3-7 ACDF with PEEK cages, PIF with C3-6 lateral mass screws and C7 pedicle screws, and PD with C3-6 laminoplasty (Fig. 2C). A bone graft was placed over the laminae of the hinge side and bilateral mass. Her JOA score improved from 12 to 17, her Nurick score decreased from 3 to 1, and her NDI decreased from 35 to 18. Her C2-7 Cobb angle improved from 13° kyphosis to 18° lordosis and her C7 slope improved from 10° lordosis to 27° lordosis. Lateral plain film 12 months postsurgery revealed a solid union over the fusion structure with no reclosure of the opened laminae from EOLP (Fig. 2D), and AP one demonstrated healing of the posterolateral fusion over the bilateral mass (Fig. 2E). MRI 12 months postsurgery revealed patent spinal canal over C3-7 (Fig. 2F).

## 4 Discussion

MCSM combined with CKD is a severe threat to a patient's spinal health and treatment requires complex surgical procedures. Patients with myelopathy and severely limited range of motion in the neck require simultaneous surgical decompression and correction. In various situations, the optimal surgical strategy is debated and the various approaches carry different risks and benefits.^[2,18]^ Through an anterior approach, restoration of C2-7 Cobb angle and disc height can be achieved.^[19]^ Posterior stabilization can provide tension band force to maintain corrected alignment, and laminoplasty can effectively decompress the long-level spinal cord and provide extra space for bone graft placement while protecting spinal cord from adhesion.^[11,12]^ In this study, we investigated two surgical methods for MCSM with CKD and reported their short-term postoperative results.

Laminoplasty is an effective method for decompression of the spinal cord^[20,21]^ and its combination with one- or two-level ACDF appears to be effective in treating coexisting MCSM and either short-segment local kyphosis or major pathology.^[9,22]^ Expansive application of EOLP in patients with MCSM combined with CKD (the ELTA method group) appeared to achieve sufficient functional recovery and a stable fusion construct 12 months postsurgery. With ELTA, ACDF could be performed from the posterior shifting of the spinal cord during EOLP, which improved safety because the spinal cord is frequently compressed both dorsally and ventrally in patients with MCSM combined with CKD.^[9,23]^

Correction of CKD is usually achieved through long-segment ACDF, which is considered a safer and easier method than posterior correction methods such as Smith–Petersen or pedicle subtraction osteotomies.^[12,19]^ PIF, with screw-plate or screw-rod systems, is often required for preventing failure or collapse of long-level anterior fusion segments (the LAPI method).^[24]^ Decompression of the spinal cord can be achieved indirectly, through anterior corrective fusion, or directly, through posterior laminectomy or laminoplasty, during PIF. We chose laminoplasty as the PD method because it can provide an additional fusion bed and prevent spinal cord adhesion.^[11]^ The patients who underwent the LAPI treatment exhibited strong restoration of the C2-7 Cobb angle, effective neck pain relief, and significant neurogenic recovery.

A comparison of the functional parameters of the two patient groups revealed that preoperative neurogenic and function conditions in the ELTA group were inferior to those in the LAPI group, whereas the VAS score for neck pain was less favorable in the LAPI group than in the ELTA group. Improvements in function, neurogenic status, and neck pain were considerable with no significant differences between groups, except for in terms of NDI. The difference in the improvement of NDI may have been due to differences in preoperative functional conditions between the groups. The preoperative degree of CKD was more severe in the LAPI group than in the ELTA group. For patients with more evident myelopathy symptoms and less severe CKD, the ELTA method appeared to be safe and effective, with motion preservation from fewer fusion segments. For patients who required a greater degree of cervical alignment correction, the LAPI method was sufficiently reliable in protecting the corrected fusion segments from collapse or failure. Women and obese patients demonstrated significantly less improvement in neck pain, and women also exhibited less improvement in NDI. Such patients may require further postoperative protection, rehabilitation, and support. A study of patients with MCSM demonstrated a significantly negative correlation between preoperative neck pain and cervical spine function, as well as between preoperative and postoperative ossified posterior longitudinal ligaments. Cervical sagittal alignment was not associated with preoperative or postoperative axial neck pain.^[25]^ In our study, the correlation between radiographic parameters and function, neurogenic, and neck pain scores was nonsignificant. However, small differences in postoperative clinical and radiographic parameters may not have been recorded as we were primarily concerned with positive surgical outcomes among patients.

Our study had some limitations. The number of patients was relatively small and the follow-up period was relatively short. Further observation of the incidence of adjacent segment disease and the strength of the corrective fusion segment is required, as are observations of functional status and evaluations of neck pain. Indications for these two methods of treating MCSM and CKD must be identified more clearly in further studies to aid spine surgeons in decision-making.

## 5 Conclusions

EOLP followed by three-segment ACDF (the ELTA method) and long-segment ACDF followed by PIF (the LAPI method) were both effective surgical strategies for treating MCSM combined with CKD based on the adequate patient outcomes. The ELTA method appears more promising for patients with MCSM with a low degree of CKD, whereas the LAPI method appears more promising for patients with MCSM with poor function scores and a high degree of CKD.

## Acknowledgment

This manuscript was edited by Wallace Academic Editing.

## Author contributions

**Conceptualization:** Wen-Tien Wu.

**Data curation:** Cheng-Huan Peng, Jen-Hung Wang, Wen-Tien Wu.

**Formal analysis:** Jen-Hung Wang.

**Investigation:** Tzai-Chiu Yu, Cheng-Huan Peng, Kuan-Lin Liu.

**Methodology:** Kuang-Ting Yeh.

**Project administration:** Ing-Ho Chen.

**Resources:** Tzai-Chiu Yu, Ing-Ho Chen.

**Supervision:** Ru-Ping Lee, Wen-Tien Wu.

**Validation:** Tzai-Chiu Yu, Wen-Tien Wu.

**Visualization:** Ru-Ping Lee, Kuan-Lin Liu, Wen-Tien Wu.

**Writing – original draft:** Kuang-Ting Yeh.

**Writing – review & editing:** Wen-Tien Wu.

Wen-Tien Wu: 0000-0001-6945-712X.

Wen-Tien Wu orcid: 0000-0001-6945-712X.

## References

[1] Tan LA Riew KD Traynelis VC Cervical spine deformity – Part 1: biomechanics, radiographic parameters, and classification. Neurosurgery 2017;81:197–203. 28838143 10.1093/neuros/nyx249

[2] Han K Lu C Li J Surgical treatment of cervical kyphosis. Eur Spine J 2011;20:523–36. 20967471 10.1007/s00586-010-1602-8 PMC3065605

[3] Morishita Y Naito M Wang JC Cervical spinal canal stenosis: the differences between stenosis at the lower cervical and multiple segment levels. Int Orthop 2011;35:1517–22. 21113592 10.1007/s00264-010-1169-3 PMC3174302

[4] Woods BI Hohl J Lee J Laminoplasty versus laminectomy and fusion for multilevel cervical spondylotic myelopathy. Clin Orthop Relat Res 2011;469:688–95. 21089002 10.1007/s11999-010-1653-5 PMC3032861

[5] Masini M Maranhão V Experimental determination of the effect of progressive sharp-angle spinal deformity on the spinal cord. Eur Spine J 1997;6:89–92. 9209874 10.1007/BF01358738 PMC3454590

[6] Passias PG Horn SR Bortz CA The relationship between improvements in myelopathy and sagittal realignment in cervical deformity surgery outcomes. Spine (Phila Pa 1976) 2018;43:1117–24. 29462071 10.1097/BRS.0000000000002610

[7] Burneikiene S Nelson EL Mason A The duration of symptoms and clinical outcomes in patients undergoing anterior cervical discectomy and fusion for degenerative disc disease and radiculopathy. Spine J 2015;15:427–32. 25264315 10.1016/j.spinee.2014.09.017

[8] Daniels AH Riew KD Yoo JU Adverse events associated with anterior cervical spine surgery. J Am Acad Orthop Surg 2008;16:729–38. 19056921 10.5435/00124635-200812000-00005

[9] Yeh KT Lee RP Chen IH Laminoplasty with adjunct anterior short segment fusion for multilevel cervical myelopathy associated with local kyphosis. J Chin Med Assoc 2015;78:364–9. 25943045 10.1016/j.jcma.2015.03.009

[10] Tang HM Yeh KT Lee RP Combined expansive open-door laminoplasty with short-segment lateral mass instrumented fusion for multilevel cervical spondylotic myelopathy with short segment instability. Tzu Chi Med J 2016;28:15–9. 10.1016/j.tcmj.2015.09.004 PMC5509173 28757711

[11] Yeh KT Lee RP Chen IH Laminoplasty instead of laminectomy as a decompression method in posterior instrumented fusion for degenerative cervical kyphosis with stenosis. J Orthop Surg Res 2015;10:138. 26338009 10.1186/s13018-015-0280-y PMC4559293

[12] Yoshihara H Abumi K Ito M Severe fixed cervical kyphosis treated with circumferential osteotomy and pedicle screw fixation using an anterior-posterior-anterior surgical sequence. World Neurosurg 2013;80:654.e17–21. 10.1016/j.wneu.2013.01.023 23313237

[13] Yeh KT Chen IH Yu TC Modified expansive open-door laminoplasty technique improved postoperative neck pain and cervical range of motion. J Formos Med Assoc 2015;114:1225–32. 25557153 10.1016/j.jfma.2014.10.005

[14] Bohm PE Fehlings MG Kopjar B Psychometric properties of the 30-m walking test in patients with degenerative cervical myelopathy: results from two prospective multicenter cohort studies. Spine J 2017;17:211–7. 27592193 10.1016/j.spinee.2016.08.033

[15] Yonenobu K Abumi K Nagata K Interobserver and intraobserver reliability of the japanese orthopaedic association scoring system for evaluation of cervical compression myelopathy. Spine (Phila Pa 1976) 2001;26:1890–4. discussion 1895. 11568701 10.1097/00007632-200109010-00014

[16] Zhu MP Tetreault LA Sorefan-Mangou F Efficacy, safety and economics of bracing after spine surgery: a systematic review of the literature. Spine J 2018;18:1513–25. 29355785 10.1016/j.spinee.2018.01.011

[17] Yeh KT Lee RP Chen IH Are there age- and sex-related differences in spinal sagittal alignment and balance among Taiwanese asymptomatic adults? Clin Orthop Relat Res 2018;476:1010–7. 29419634 10.1007/s11999.0000000000000140 PMC5916630

[18] Grosso MJ Hwang R Krishnaney AA Complications and outcomes for surgical approaches to cervical kyphosis. J Spinal Disord Tech 2015;28:E385–93. 23732179 10.1097/BSD.0b013e318299953f

[19] Zdeblick TA Bohlman HH Cervical kyphosis and myelopathy. Treatment by anterior corpectomy and strut-grafting. J Bone Joint Surg Am 1989;71:170–82. 2645290

[20] Kaplan L Bronstein Y Barzilay Y Canal expansive laminoplasty in the management of cervical spondylotic myelopathy. Isr Med Assoc J 2006;8:548–52. 16958245

[21] Yeh KT Yu TC Chen IH Expansive open-door laminoplasty secured with titanium miniplates is a good surgical method for multiple-level cervical stenosis. J Orthop Surg Res 2014;9:49. 25142174 10.1186/s13018-014-0049-8 PMC4237882

[22] Baba H Furusawa N Imura S Laminoplasty following anterior cervical fusion for spondylotic myeloradiculopathy. Int Orthop 1994;18:1–5. 8021059 10.1007/BF00180168

[23] Liu XY Yuan SM Tian YH Expansive open-door laminoplasty and selective anterior cervical decompression and fusion for treatment of multilevel cervical spondylotic myelopathy. Orthop Surg 2011;3:161–6. 22009646 10.1111/j.1757-7861.2011.00143.x PMC6583147

[24] Kawabata S Watanabe K Hosogane N Surgical correction of severe cervical kyphosis in patients with neurofibromatosis Type 1. J Neurosurg Spine 2013;18:274–9. 23289507 10.3171/2012.11.SPINE12417

[25] Fujiwara H Oda T Makino T Impact of cervical sagittal alignment on axial neck pain and health-related quality of life after cervical laminoplasty in patients with cervical spondylotic myelopathy or ossification of the posterior longitudinal ligament: a prospective comparative study. Clin Spine Surg 2018;31:E245–51. 29481340 10.1097/BSD.0000000000000619

